# Supplementary material for: Video-rate 3D imaging of living cells using Fourier view-channel-depth light field microscopy
Source: Commun Biol. 2023 Dec 12;6:1259. doi: 10.1038/s42003-023-05636-x (PMC10716377; doi:10.1038/s42003-023-05636-x)
Supplement: Supplementary file 2 — Supplementary Materials: Video-rate 3D live-cell imaging with Fourier view-channel-depth light field microscopy [file 42003_2023_5636_MOESM2_ESM.pdf]

Supplementary Materials: Video-rate 3D live-cell imaging with Fourier view-channel-depth light field microscopy

Chengqiang Yi,[1#] Lanxin Zhu,[1#] Jiahao Sun,[1] Zhaofei Wang,[1] Meng Zhang,[1,2] Fenghe Zhong, [1] Luxin Yan,[3] Jiang Tang,[1] Liang Huang,[4] Yu-Hui Zhang,[2] Dongyu Li,[1*] Peng Fei,[1]

[1]School of Optical and Electronic Information-Wuhan National Laboratory for Optoelectronics-Advanced Biomedical Imaging Facility, Huazhong University of Science and Technology, Wuhan, China, 430074

[2]Britton Chance Center for Biomedical Photonics-MoE Key Laboratory for Biomedical Photonics, Advanced Biomedical Imaging Facility-Wuhan National Laboratory for Optoelectronics, Huazhong University of Science and Technology, Wuhan, Hubei, China, 430074

[3]State Education Commission Key Laboratory for Image Processing and Intelligent Control, Huazhong University of Science and Technology, Wuhan, Hubei, China, 430074

[4]Department of Hematology, Tongji Hospital, Tongji Medical College, Huazhong University of Science and Technology, Wuhan, Hubei 430030

Keywords: Fourier light field microscopy, living cell imaging, view-channel-depth (VCD) reconstruction, intracellular dynamics, three-dimensional microscopy.

[#] These authors contributed equally to this work.

[*] Correspondence：li_dongyu@hust.edu.cn

Supplementary Figures

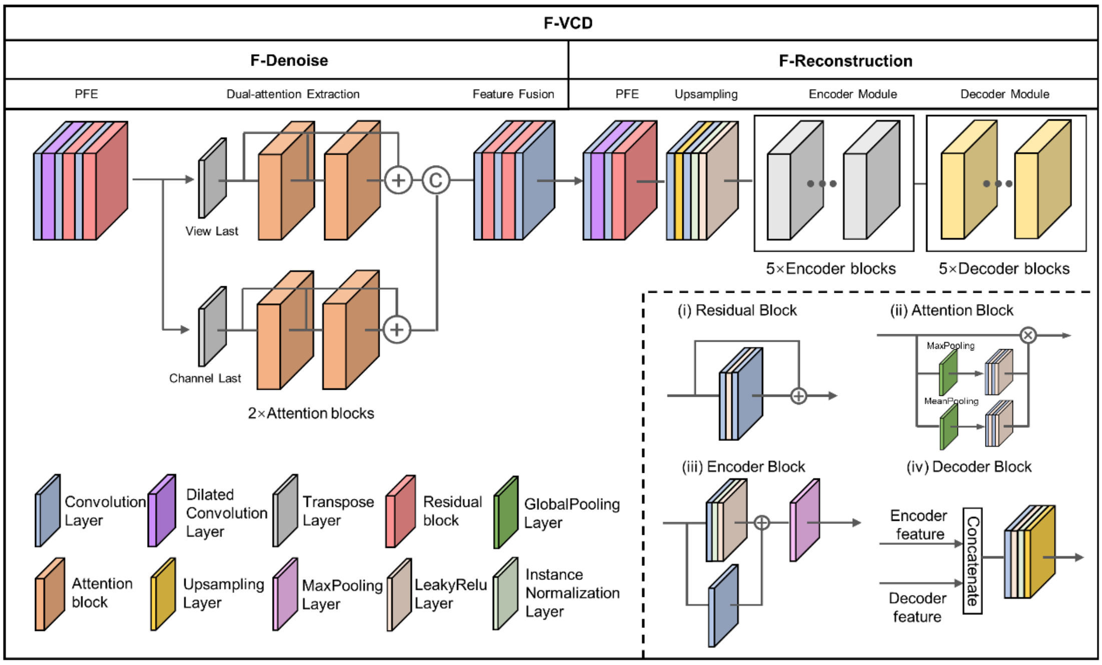

Supplementary Figure 1: The overview of F-VCD structure. F-VCD is composed of two modules: "F-Denoise" and "F-Reconstruction". The first part "F-Denoise" is composed of primary feature extraction (PFE), dual-attention extraction branch and feature fusion blocks. For PFE module, we use three kinds of dilation convolution (rate=1,2,4) and residual blocks to achieve feature extraction. The number of filters is set as 32, which converted the input view stack (dimensions $1\times h\times w\times 3$, batch×height × width×views) into output features (dimensions $3\times h\times w\times 32$, batch×height × width×channel). Then, we transpose the order of feature dimensions to get two types of data, "view domain" and "channel domain" and feed into a RCAN-based dual-attention module. In the attention block of this module, we use both MaxPooling and MeanPooling operations to accumulate global information. After the dual-attention module, a concatenation layer and two residual blocks fuse the extracted features. According to 1×1 convolution， we descend the dimensions of outputs and get denoised view stack. In the F-Reconstruction module, based on our previous VCD network, we add three dilation convolution blocks to extract primary features and use one subpixel layer to upscale the lateral size of extracted features. The number of base filters is set to 126. For the better convergence of the module, we replace the plain 2D convolution into the residual block in the "Encoder" part of VCD, which convert the primary features (dimensions $1\times h\times w\times 126$) into a super-resolution 3D reconstruction (dimensions $1\times s{\cdot}h\times s{\cdot}w\times depth$, batch×height × width×depth). In this paper, we set the super-resolution factor s as 3 and $depth$ as 41.

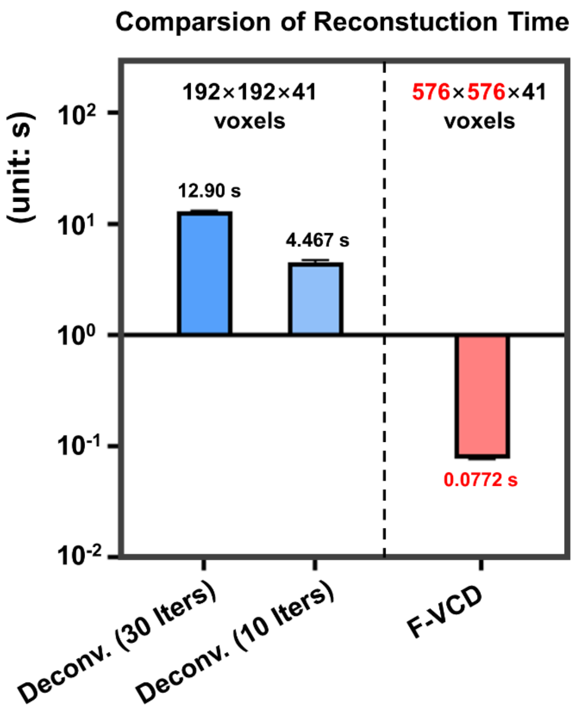

Supplementary Figure 2: The computational cost comparison between deconvolution and F-VCD. DL-based F-VCD achieved two-orders of magnitude acceleration and produced 9 times number of voxels compared with deconvolution. The speed comparison was tested on RTX 3090.

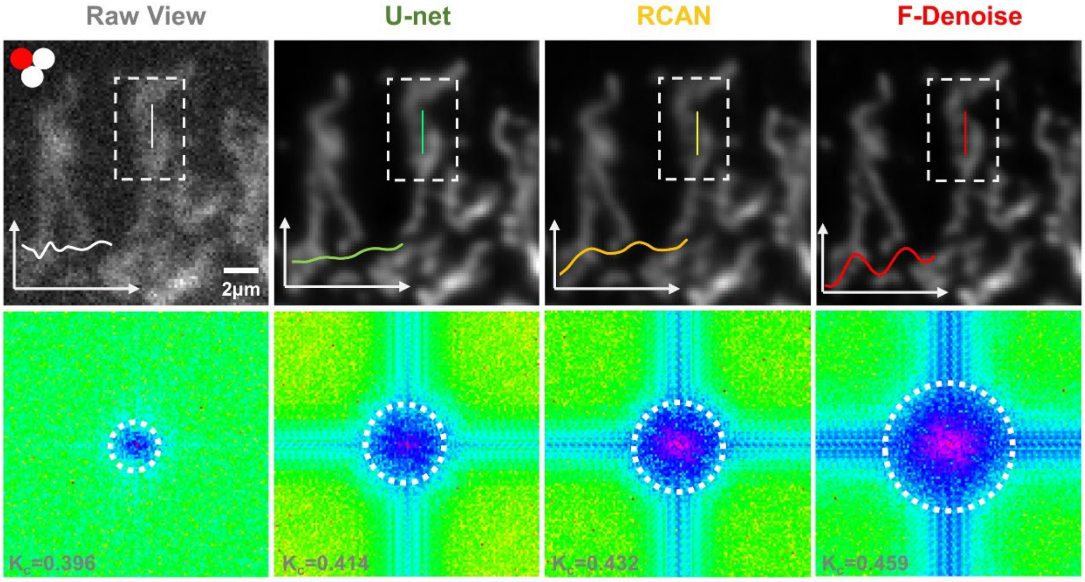

Supplementary Figure 3: Comparison of different denoising networks on noisy input FLFM views. Living U2OS cells were imaged using FLFM at low excitation power. The top panel shows a noisy view of the raw FLFM image and its denoised results by different networks. The bottom panel shows the frequency spectrum corresponding to each image in the upper panel. The measured K_c (cutoff-frequency) metrics indicate that F-denoise module is superior to other networks.

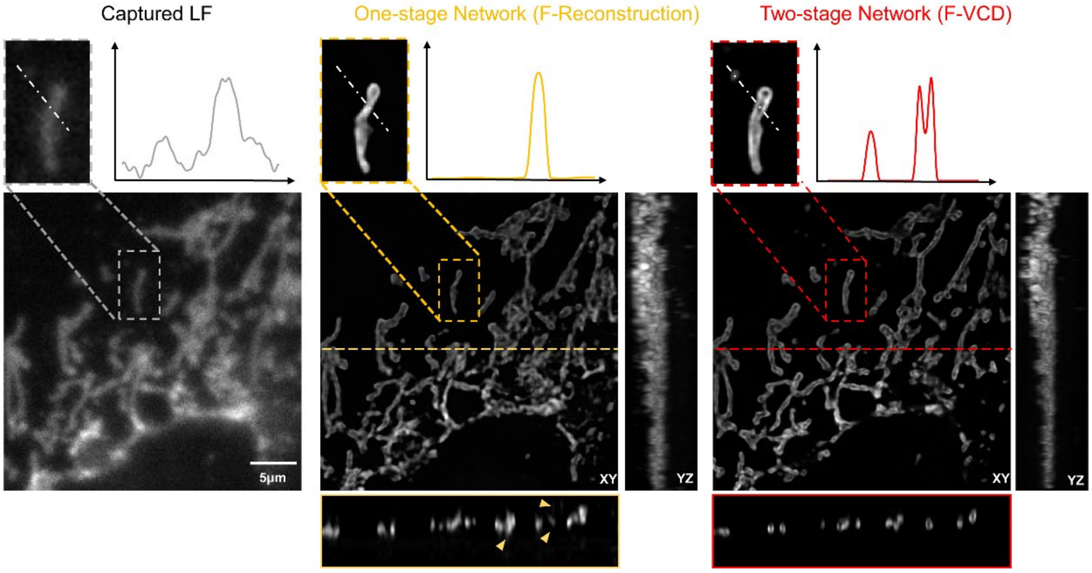

Supplementary Figure 4: Comparison of the one-step network (F-Reconstruction) and a two-step one (F-VCD) on 3D FLFM reconstruction of living mitochondrial. F-VCD reconstruction shows improved resolution and reduced artifacts with the extra F-Denoising module.

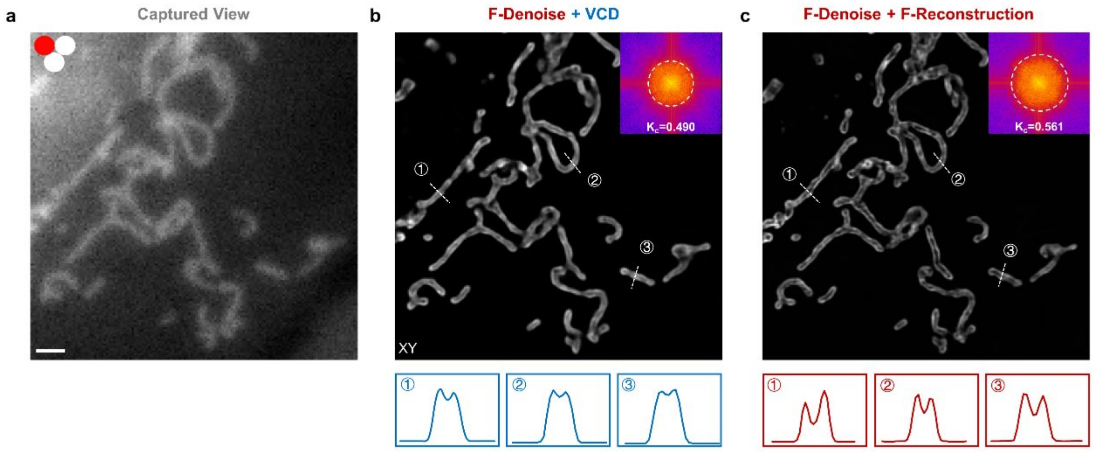

Supplementary Figure 5: Comparison between VCD and F-Reconstruction module. a one of the captured triple views. **b** MIP of "F-Denoise + VCD" reconstruction from noisy views. **c** MIP of "F-Denoise + F-Reconstruction" reconstruction from noisy views, which shows higher resolution and contrast than conventional VCD indicated by the plots of ROI and measured K_c. Scale bar: 2 μm

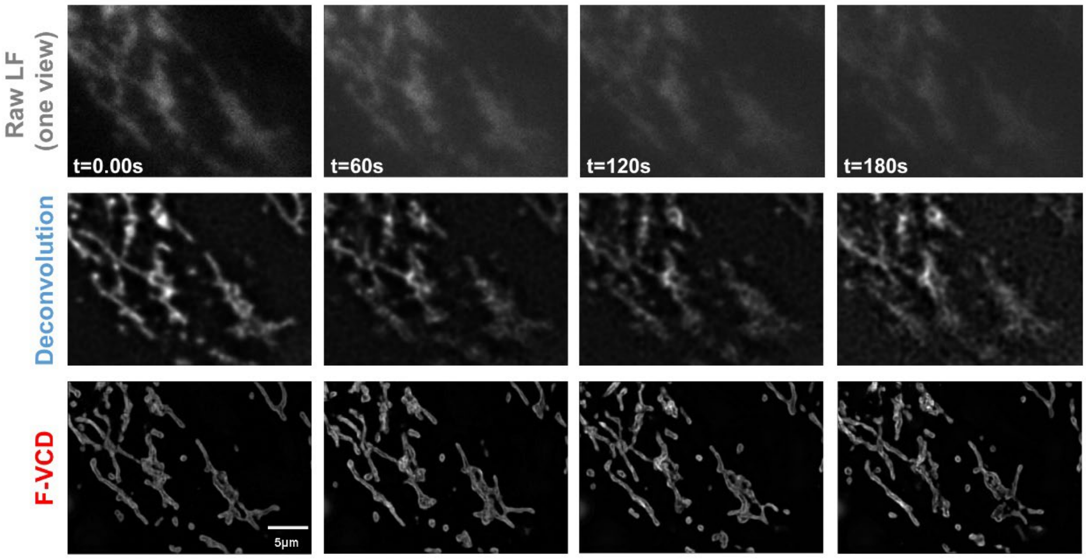

Supplementary Figure 6: Comparison of F-VCD and deconvolution-based algorithm on 3D FLFM reconstruction of living mitochondrial during sustained imaging. When the SNR of captured LF decreases, the deconvolution-based results are dramatically corrupted with the severe ringing artifacts and blurring signals while F-VCD reconstruction performs high robustness for this noise degradation.

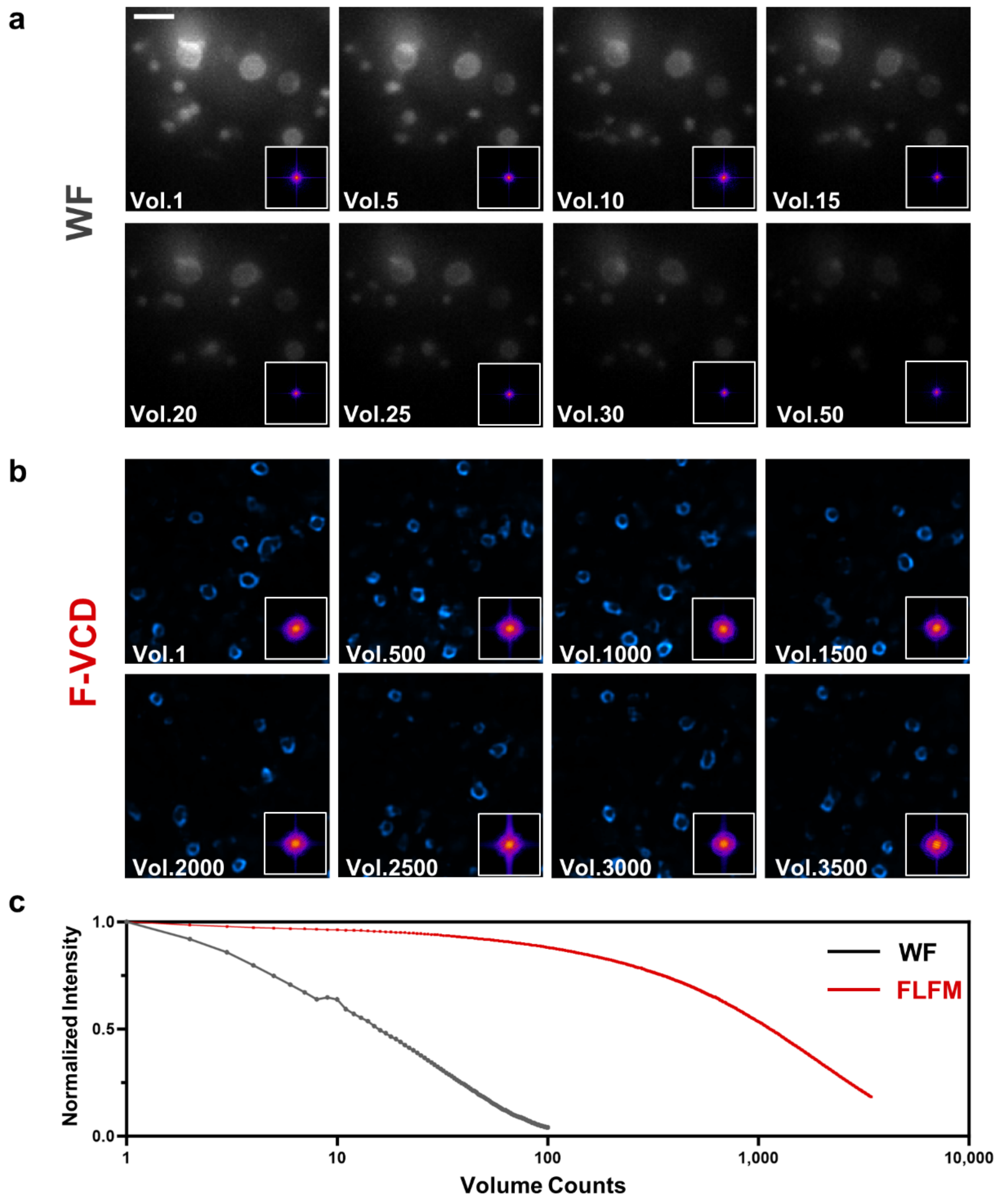

Supplementary Figure 7: The robustness of F-VCD under low photon budget assayed by long-term living cell imaging. a Wide-field scanning imaging for the living U2OS cell with lysosome. Due to the low efficiency of photon-utilization of WF, the repeat excitation by sequential scanning produce dramatical photobleaching denoted by the intensity decrease of the 50th Vol and frequency bandwidth reduction. Scale bar: 2μm. **b** F-VCD reconstruction from captured 2D FLFM images. With the denoising ability and resolution enhancement of our network, the reconstruction results perform detailed structure reconstruction (the ring-like structure of lysosome outer membrane) until the 3500th volume. **c** The normalized intensity plots of WF and FLFM captures, which shows the snapshot volumetric imaging of FLFM outperforms the scanning-based approaches in long-term imaging due to its high photon efficiency.

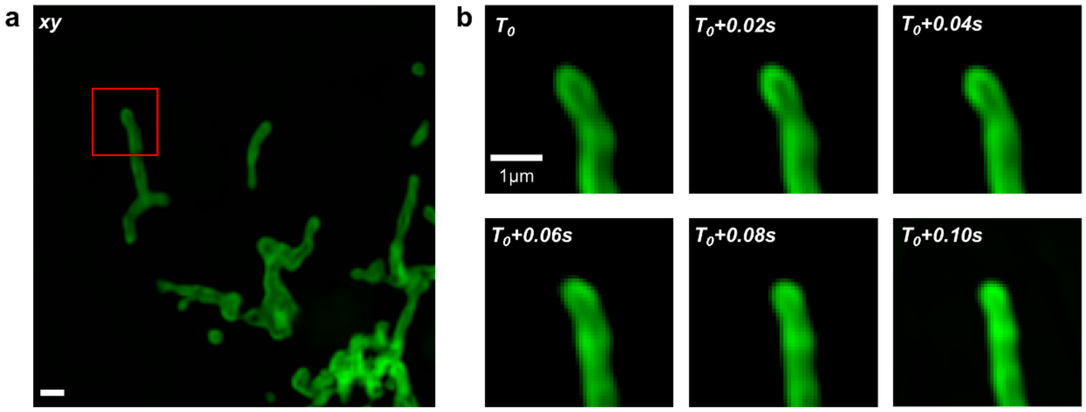

Supplementary Figure 8: F-VCD reconstruction of local wiggling of mitochondria in a living cell. a The lateral projection of F-VCD reconstruction at interval time of 20 ms. **b** The higher magnification views show the locomotion of mitochondria.

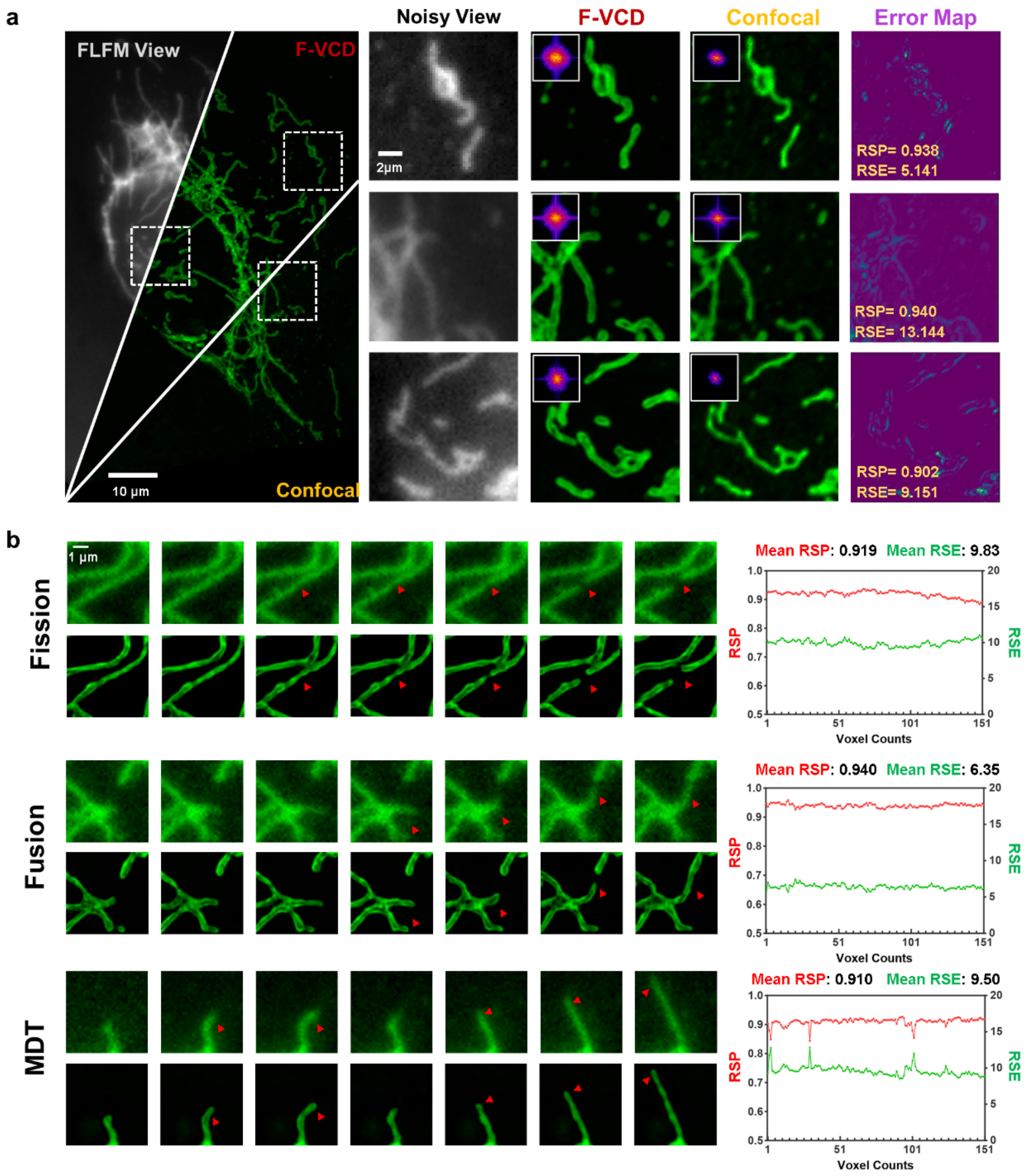

Supplementary Figure 9: Quantitative validation of F-VCD reconstruction. a The *in situ* imaging comparison between confocal microscopy and F-VCD reconstruction via FLFM on fixed U2OS cell with mitochondria. F-VCD results perform higher contrast and detailed structure. The high RSP (above 0.9) and low RSE (~10) denote F-VCD's comparable accuracy with conventional confocal imaging. **b** Quantitative evaluation of dynamic reconstruction. *Left*: Represent images of captured view and corresponding F-VCD reconstruction during the time window of dynamic events. The red arrows denote the local morphology of mitochondria. *Right*: The statistical results of error evaluation in living cell imaging. The mean RSP and RSE of F-VCD reconstruction show the similar accuracy across the dynamic events.

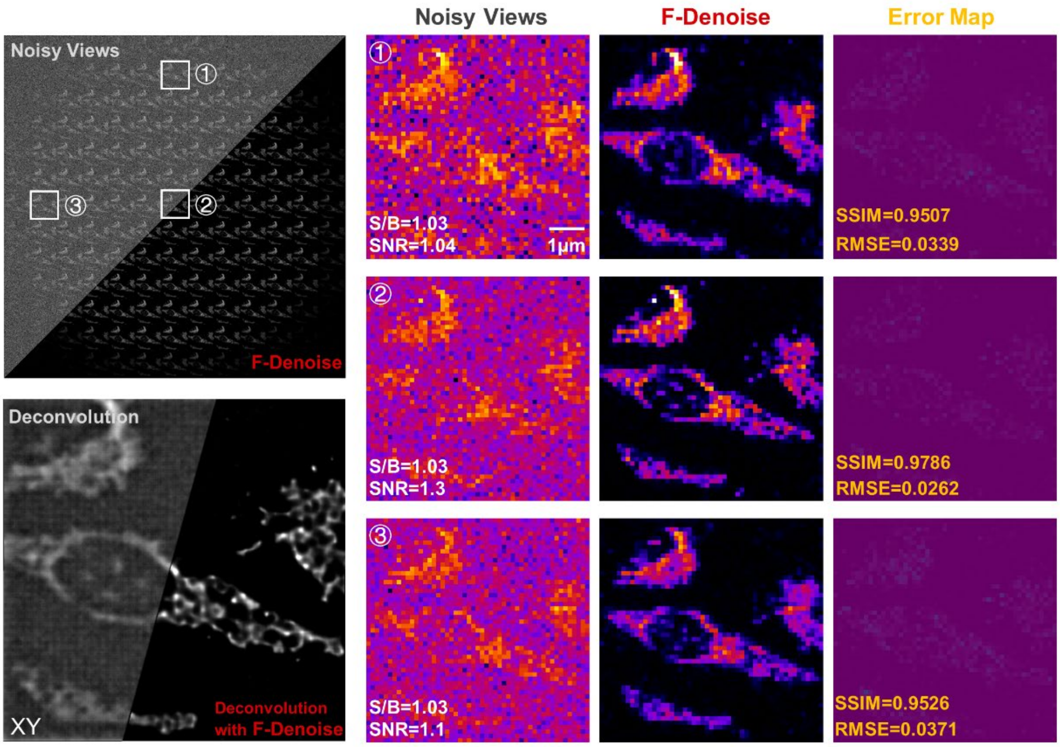

Supplementary Figure 10: The compatibility of F-Denoise validated on conventional LFM. With the view-attention denoising module, F-Denoise can be well implemented on conventional LFM. The noisy multi-views of simulated LFM were enhanced by F-Denoise with high SSIM (Right), which also promoted the artifacts suppression and background removal for latter 3D deconvolution reconstruction.
