# Supplementary material for: Video-rate 3D imaging of living cells using Fourier view-channel-depth light field microscopy
Source: Commun Biol. 2023 Dec 12;6:1259. doi: 10.1038/s42003-023-05636-x (PMC10716377; doi:10.1038/s42003-023-05636-x)
Supplement: Supplementary file 3 — Description of Additional Supplementary Files [file 42003_2023_5636_MOESM3_ESM.docx]

**Description of Additional Supplementary Files**

**File name: Supplementary Video 1**

**Description: F-VCD enables long-term living cell imaging**

**File name: Supplementary Video 2**

**Description: F-VCD enables capturing dynamic events**

**File name: Supplementary data 1**

**Description: This file contains the source data for all graphs and charts in the main and supplementary figures.**
