# Supplementary material for: Video-rate 3D imaging of living cells using Fourier view-channel-depth light field microscopy
Source: Commun Biol. 2023 Dec 12;6:1259. doi: 10.1038/s42003-023-05636-x (PMC10716377; doi:10.1038/s42003-023-05636-x)
Supplement: Supplementary file 7 — Reporting Summary [file 42003_2023_5636_MOESM7_ESM.pdf]

nature portfolio

Corresponding author(s): Dongyu Li

Last updated by author(s): Nov 13, 2023

Reporting Summary

Nature Portfolio wishes to improve the reproducibility of the work that we publish. This form provides structure for consistency and transparency in reporting. For further information on Nature Portfolio policies, see our Editorial Policies and the Editorial Policy Checklist.

Statistics

For all statistical analyses, confirm that the following items are present in the figure legend, table legend, main text, or Methods section.

n/a	Confirmed	
☐	☒	The exact sample size (*n*) for each experimental group/condition, given as a discrete number and unit of measurement
☐	☒	A statement on whether measurements were taken from distinct samples or whether the same sample was measured repeatedly
☐	☒	The statistical test(s) used AND whether they are one- or two-sided<br>*Only common tests should be described solely by name; describe more complex techniques in the Methods section.*
☒	☐	A description of all covariates tested
☐	☒	A description of any assumptions or corrections, such as tests of normality and adjustment for multiple comparisons
☐	☒	A full description of the statistical parameters including central tendency (e.g. means) or other basic estimates (e.g. regression coefficient) AND variation (e.g. standard deviation) or associated estimates of uncertainty (e.g. confidence intervals)
☒	☐	For null hypothesis testing, the test statistic (e.g. *F*, *t*, *r*) with confidence intervals, effect sizes, degrees of freedom and *P* value noted<br>*Give P values as exact values whenever suitable.*
☒	☐	For Bayesian analysis, information on the choice of priors and Markov chain Monte Carlo settings
☐	☒	For hierarchical and complex designs, identification of the appropriate level for tests and full reporting of outcomes
☒	☐	Estimates of effect sizes (e.g. Cohen's *d*, Pearson's *r*), indicating how they were calculated

Our web collection on statistics for biologists contains articles on many of the points above.

Software and code

Policy information about availability of computer code

Data collection	The original light-field imaging data were collected using our customized FLFM microscopy by open source software Mciro-Manager. The high-resolution cell data for network training and validation were collected by confocal microscope (ZEISS LSM 980 with Airyscan 2 and OLYMPUS IXplore SpinSR).
Data analysis	Deep learning models reported in this network were built using Tensorflow 1.12.0, Tensorlayer 1.8.1 and python 3. The source code of F-VCD was uploaded into GitHub(https://github.com/feilab-hust/F-VCD). The statistical evaluation of proposed methods and analysis of biological data were performed with Matlab 2020b, Fiji, Imaris 7.5.1 and GraphPad Prism

For manuscripts utilizing custom algorithms or software that are central to the research but not yet described in published literature, software must be made available to editors and reviewers. We strongly encourage code deposition in a community repository (e.g. GitHub). See the Nature Portfolio guidelines for submitting code & software for further information.

Data

Policy information about availability of data

All manuscripts must include a data availability statement. This statement should provide the following information, where applicable:

- Accession codes, unique identifiers, or web links for publicly available datasets
- A description of any restrictions on data availability
- For clinical datasets or third party data, please ensure that the statement adheres to our policy

The data supporting the findings of this study are available within the article, the Supplementary Information files and the Source Data files that accompany this article. Source Data are provided with this paper.

Research involving human participants, their data, or biological material

Policy information about studies with human participants or human data. See also policy information about sex, gender (identity/presentation), and sexual orientation and race, ethnicity and racism.

Reporting on sex and gender	*Use the terms sex (biological attribute) and gender (shaped by social and cultural circumstances) carefully in order to avoid confusing both terms. Indicate if findings apply to only one sex or gender; describe whether sex and gender were considered in study design; whether sex and/or gender was determined based on self-reporting or assigned and methods used. Provide in the source data disaggregated sex and gender data, where this information has been collected, and if consent has been obtained for sharing of individual-level data; provide overall numbers in this Reporting Summary. Please state if this information has not been collected. Report sex- and gender-based analyses where performed, justify reasons for lack of sex- and gender-based analysis.*
Reporting on race, ethnicity, or other socially relevant groupings	*Please specify the socially constructed or socially relevant categorization variable(s) used in your manuscript and explain why they were used. Please note that such variables should not be used as proxies for other socially constructed/relevant variables (for example, race or ethnicity should not be used as a proxy for socioeconomic status). Provide clear definitions of the relevant terms used, how they were provided (by the participants/respondents, the researchers, or third parties), and the method(s) used to classify people into the different categories (e.g. self-report, census or administrative data, social media data, etc.) Please provide details about how you controlled for confounding variables in your analyses.*
Population characteristics	*Describe the covariate-relevant population characteristics of the human research participants (e.g. age, genotypic information, past and current diagnosis and treatment categories). If you filled out the behavioural & social sciences study design questions and have nothing to add here, write "See above."*
Recruitment	*Describe how participants were recruited. Outline any potential self-selection bias or other biases that may be present and how these are likely to impact results.*
Ethics oversight	*Identify the organization(s) that approved the study protocol.*

Note that full information on the approval of the study protocol must also be provided in the manuscript.

Field-specific reporting

Please select the one below that is the best fit for your research. If you are not sure, read the appropriate sections before making your selection.

☒ Life sciences ☐ Behavioural & social sciences ☐ Ecological, evolutionary & environmental sciences

For a reference copy of the document with all sections, see nature.com/documents/nr-reporting-summary-flat.pdf

Life sciences study design

All studies must disclose on these points even when the disclosure is negative.

Sample size	For F-VCD training, the size of training dataset was not predetermined based on statistical calculations. The number of training pairs (mitochondria && endoplasmic reticulum && lysosome) was usually around 1500. The number of samples acquired for training has been determined by model performance (extra data would be further acquired until model reached satisfactory capability).
Data exclusions	No data were excluded from the data analysis.
Replication	The resolution of deconvolution and F-VCD in Figure 3.c and Figure 5.f were measured for 10 different regions. The computation time of F-VCD and deconvolution in Supplementary Figure 2 were measured at least 20 times repeatedly. Living cell imaging has been repeated at least 5 times and the results were shown in Figure 5. b, Figure 5. c, Figure 5. d, Supplementary Figure 4, Supplementary Figure 7 and Supplementary Figure 8. The detailed statistical data were included in supplementary information.
Randomization	The samples were randomly chosen before imaging experiments. Training, validation and testing dataset for the network model have been randomly generated.

Blinding	All the performance evaluation of F-VCD model has been blindly tested on data that were not included in the training or validation process.

Reporting for specific materials, systems and methods

We require information from authors about some types of materials, experimental systems and methods used in many studies. Here, indicate whether each material, system or method listed is relevant to your study. If you are not sure if a list item applies to your research, read the appropriate section before selecting a response.

Materials & experimental systems

n/a	Involved in the study
☐	☒ Antibodies
☐	☒ Eukaryotic cell lines
☒	☐ Palaeontology and archaeology
☒	☐ Animals and other organisms
☒	☐ Clinical data
☒	☐ Dual use research of concern
☒	☐ Plants

Methods

n/a	Involved in the study
☒	☐ ChIP-seq
☒	☐ Flow cytometry
☒	☐ MRI-based neuroimaging

Antibodies

Antibodies used	Primary antibody: mouse anti-alpha tubulin antibody, abcam catab7291), was used 1:500 dilution; rabbit anti-vimentin antibodyabcam (cat#ab92547), was used 1:500 dilution; rabbit anti-nup 133 antibody, abcam (cat#ab155990), was used 1:100 dilution.Secondary antibody: Alexa Fluor 488 conjugated goat anti mouse antibody, abcam (cat#ab150113), was used 1:400 dilution; AlexaFluor 488 conjugated goat anti rabbit antibody, abcam (cat#ab150077), was used 1:400 dilution.
Validation	All the antibodies used in this study were validated by the vendor abcam and have been extensively used in previous studies. These labels include:<br>mouse anti-alpha tubulin, abcam (cat#a): Huang Zet al.Science Advances(2021)<br>https://www.abcam.cn/alpha-tubulin-antibodv-dmla-loading-control-ab7291.html<br>rabbit anti-vimentin,abcam (cat#ab92547):Tian (et al.Nature Communications(2021)<br>https://www.abcam.cn/vimentin-antibod-epr3776-cvtoskeleton-marker-ab92547.html<br>rabbit anti-nup 133, abcam (catab155990): Liu S.et al.Nature (2018).<br>https://wwwabcamcn/nup133-antibody-epr10808b-ab155990.html<br>Alexa Fluor 488 conjugated goat anti mouse antibody, abcam (cat#ab150113):<br>Tulpule A. et al. Cell (2021).<br>https://wwwabcamcn/goat-mouse-igg-hl-alexa-fluor-488-ab150113.htm<br>Alexa Fluor 488 conjugated goat anti rabbit antibody, abcam (cat#ab150077):<br>Zhang C.et al Nature Communications (2021)<br>https://wwwabcamcn/goat-rabbit-igg-hl-alexa-fluor-488-ab150077.htm

Eukaryotic cell lines

Policy information about cell lines and Sex and Gender in Research

Cell line source(s)	U20S cells were purchased from Procell Life Science & Technology Co, Ltd. (Wuhan, Hubei, China)
Authentication	The cell lines were authenticated by Procell Life Science & Technology, and were not specifically authenticated in my lab.
Mycoplasma contamination	Cell lines used in this study tested negative for mycoplasma contamination.
Commonly misidentified lines (See ICLAC register)	There were no misidentified cell lines used in this study

Plants

Seed stocks	*Report on the source of all seed stocks or other plant material used. If applicable, state the seed stock centre and catalogue number. If plant specimens were collected from the field, describe the collection location, date and sampling procedures.*
Novel plant genotypes	*Describe the methods by which all novel plant genotypes were produced. This includes those generated by transgenic approaches, gene editing, chemical/radiation-based mutagenesis and hybridization. For transgenic lines, describe the transformation method, the number of independent lines analyzed and the generation upon which experiments were performed. For gene-edited lines, describe the editor used, the endogenous sequence targeted for editing, the targeting guide RNA sequence (if applicable) and how the editor was applied.*
Authentication	*Describe any authentication procedures for each seed stock used or novel genotype generated. Describe any experiments used to assess the effect of a mutation and, where applicable, how potential secondary effects (e.g. second site T-DNA insertions, mosiacism, off-target gene editing) were examined.*
